# Production of reactive oxygen species and wound-induced resistance in *Arabidopsis thaliana* against *Botrytis cinerea* are preceded and depend on a burst of calcium

**DOI:** 10.1186/1471-2229-13-160

**Published:** 2013-10-17

**Authors:** Emna Beneloujaephajri, Alex Costa, Floriane L’Haridon, Jean-Pierre Métraux, Matteo Binda

**Affiliations:** 1 Department of biology, University of Fribourg, Ch. du Musée 10, 1700 Fribourg, Switzerland; 2 Department of Biosciences, University of Milan, via G. Celoria 26, 20133 Milan, Italy; 3 Current address: Medion Grifols Diagnostics AG, Bonnstrasse 9, 3186 Düdingen, Switzerland

**Keywords:** *Arabidopsis thaliana*, *Botrytis cinerea*, Cuticle, Wounding, Resistance, Calcium, ROS

## Abstract

**Background:**

Wounded leaves of *Arabidopsis thaliana* produce *r*eactive *o*xygen *s*pecies (ROS) within minutes after wounding and become resistant to the pathogenic fungus *Botrytis cinerea* at a local level. This fast response of the plants to the wound is called *w*ound-*i*nduced *r*esistance (WIR). However the molecular mechanisms of this response and the signal cascade between the wound and ROS production are still largely unknown. Calcium is a conserved signal and it is involved in many abiotic stress responses in plants, furthermore, calcium pathways act very fast.

**Results:**

The results of this study show that leaves treated with calcium channels inhibitors (verapamil) or calcium chelators (oxalate and EGTA) are impaired in ROS production. Moreover, leaves treated with verapamil, EGTA or oxalate were more susceptible to *B. cinerea* after wounding. The intracellular measurements of calcium changes indicated quick but transient calcium dynamics taking place few seconds after wounding in cells neighbouring the wound site. This change in the cytosolic calcium was followed in the same region by a more stable ROS burst.

**Conclusions:**

These data further extend our knowledge on the connection between wounding, calcium influx and ROS production. Moreover they provide for the first time the evidence that, following wounding, calcium changes precede a burst in ROS in the same location.

## Background

Damage of the cuticle, a hydrophobic layer protecting the aerial parts of plants, results in a loss of water and facilitates the entrance of various pathogens. This textbook statement has to be somewhat nuanced in the light of recent findings. For instance, when leaves of bean or *Arabidopsis thaliana* plants are treated ectopically with fungal cutinase, they display resistance to fungal pathogens [1,2]. Furthermore, *A. thaliana* engineered to express a fungal cutinase targeted to the cell wall also express a strong resistance [2]. The increased cuticular permeability allows for a better perception of elicitors generated during infection [3]. To further explore these observations, leaf surfaces of Arabidopsis were wounded as an alternative approach to modify the cuticular barrier. This resulted in complete immunity to *B. cinerea*[4]. This paradoxical finding was eventually explained when it was observed that this wound-induced resistance was only expressed when plants were maintained at high humidity after wounding. Furthermore, ROS including H_2_O_2_ and O_2_^-^ can be detected within minutes at the wound sites of *A. thaliana* leaves crushed with forceps. ROS precede and seem causally related with full immunity to *B. cinerea* inoculated at the wounded areas [5]. Indeed, wound-induced ROS are not detected and resistance to *B. cinerea* is lost in plants incubated under dry conditions after wounding [5]. The stress hormone *ab*scisic *a*cid (ABA) is strongly linked to the loss of ROS under dry conditions. ABA accumulates within 90 minutes after wounding in dry conditions while mutants damaged in ABA synthesis still produce ROS and display strong resistance under the same conditions [5]. Interestingly, mutants with an increased cuticular permeability exhibit a similar high production of ROS that is associated with complete immunity [5]. These experiments naturally lead to the question why the plant fails to react to the tissue damage inflicted by *B. cinerea* under natural situations. Under natural conditions a weak ROS production is only observed late after inoculation with *B. cinerea* (ca 12 h after inoculation) despite breakdown of the cuticle by the fungus. This question was eventually clarified when it was shown that the fungus interferes with ROS accumulation by releasing oxalic acid, a calcium chelator, at the infection site [5]. The possible interference of oxalate produced by *B. cinerea* with calcium makes it likely that calcium is involved in ROS formation and in the subsequent WIR. Another example for the interference with calcium signalling exists in phytopathogenic bacteria that produce polysaccharides that act at the same time as *m*icrobial *a*ssociated *m*olecular *p*atterns (MAMPs) and virulence factors with a calcium-chelating action [6].

In this work, we have investigated the hypothesis that calcium is involved in ROS accumulation and WIR to *B. cinerea* in leaves of *A. thaliana*. Our experiments indicate that changes in calcium are relevant and take place at the same localization but earlier than ROS in wounded leaves.

## Results

### Application on leaves of molecules disturbing calcium fluxes prevents ROS production after wounding and WIR

**Figure 1:**
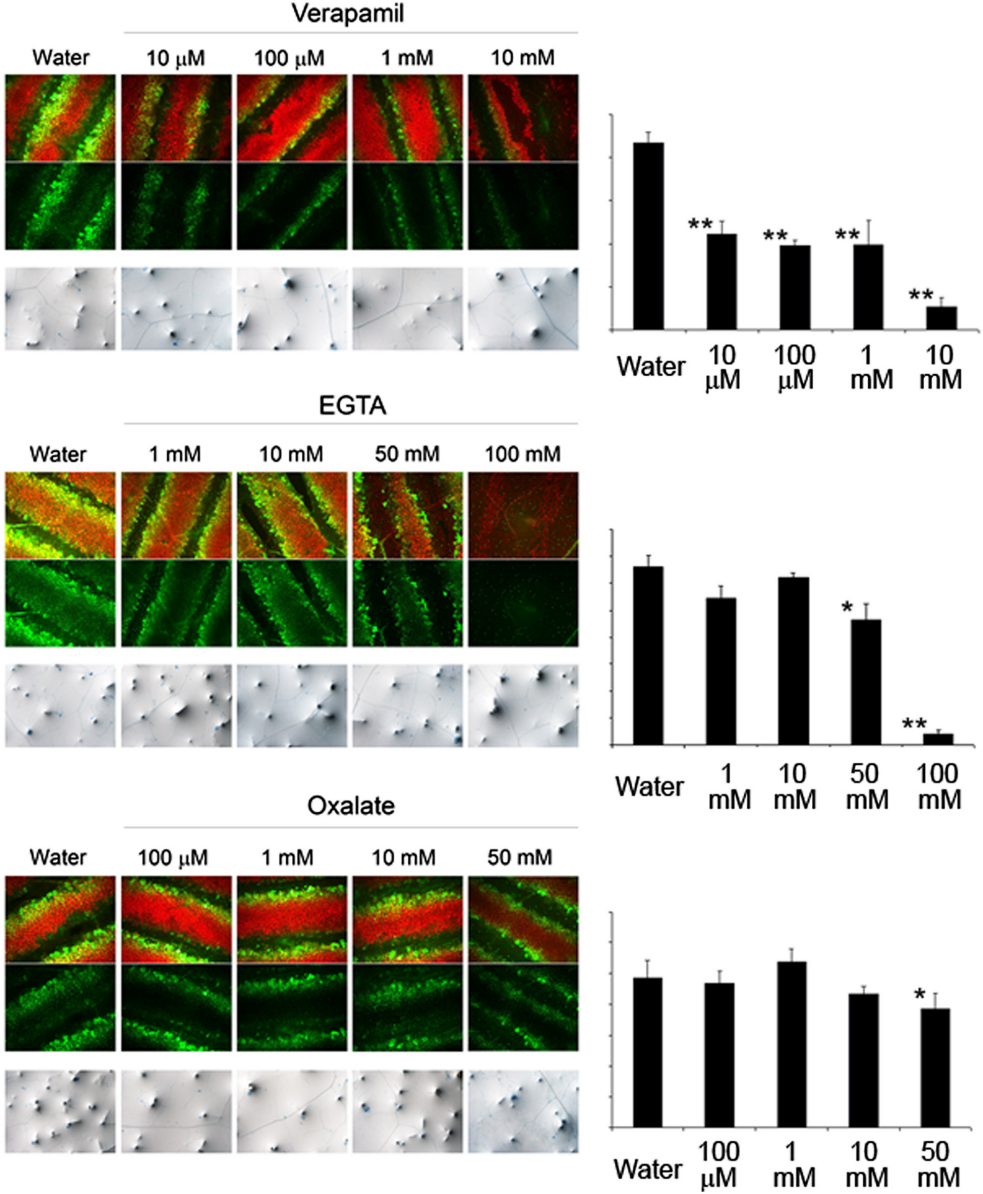
**Treatment of *A. thaliana *leaves with calcium channel blocker and calcium chelators abolishes ROS production after leaves wounding.** Droplets containing different concentrations of verapamil (10 μM, 100 μM, 1 mM, 10 mM), EGTA (1 mM, 10 mM, 50 mM, 100 mM) and oxalate (100 μM, 1 mM, 10 mM, 50 mM) were applied on leaves for 3 hours. Leaves were then either wounded and stained with DCF-DA and visualized at a fluorescence microscope with two sets of filters (ROS in green and chlorophyll autofluorescence in red, upper panels), or were tested for cell viability using trypan blue (lower panels). Densitometric analysis of the ROS signal is displayed on the right of each image series. Asterisks represent significant differences using Student's *t* test relative to water-treated control; *P < 0.05, **P < 0.01.

Wounded *A. thaliana* leaves generate ROS within minutes as revealed after infiltration with the 5-(and-6)-carboxy-2,7-*d*i*c*hlorodihydro*f*luorescein*d*i*a*cetate (DCF-DA) probe [5] (Figure 1). We have used a pharmacological approach to examine the importance of calcium in wound-induced ROS formation. A calcium channel blocker (verapamil) or calcium chelators (EGTA or oxalate) were applied as small droplets on the upper side of Arabidopsis leaves. After 3 hours the droplets were removed and leaves were wounded at those sites. Verapamil (starting at 10 μM), EGTA (starting at 50 mM) and oxalate (starting at 50 mM) all affected wound induced ROS formation (Figure 1). Using this way of application, we cannot know how much of these compounds penetrated in the extracellular space. The effects of the chelators were visible at concentrations in the same range of the estimate extracellular calcium concentration (1–10 mM) [7]. A dose–response effect of the compounds tested could be detected starting at lower concentrations only when applied by vacuum-infiltration of detached leaves prior to wounding (Additional file 1). None of these compounds affected cell vitality at the concentrations used as determined using trypan blue tests (Figure 1).

**Figure 2:**
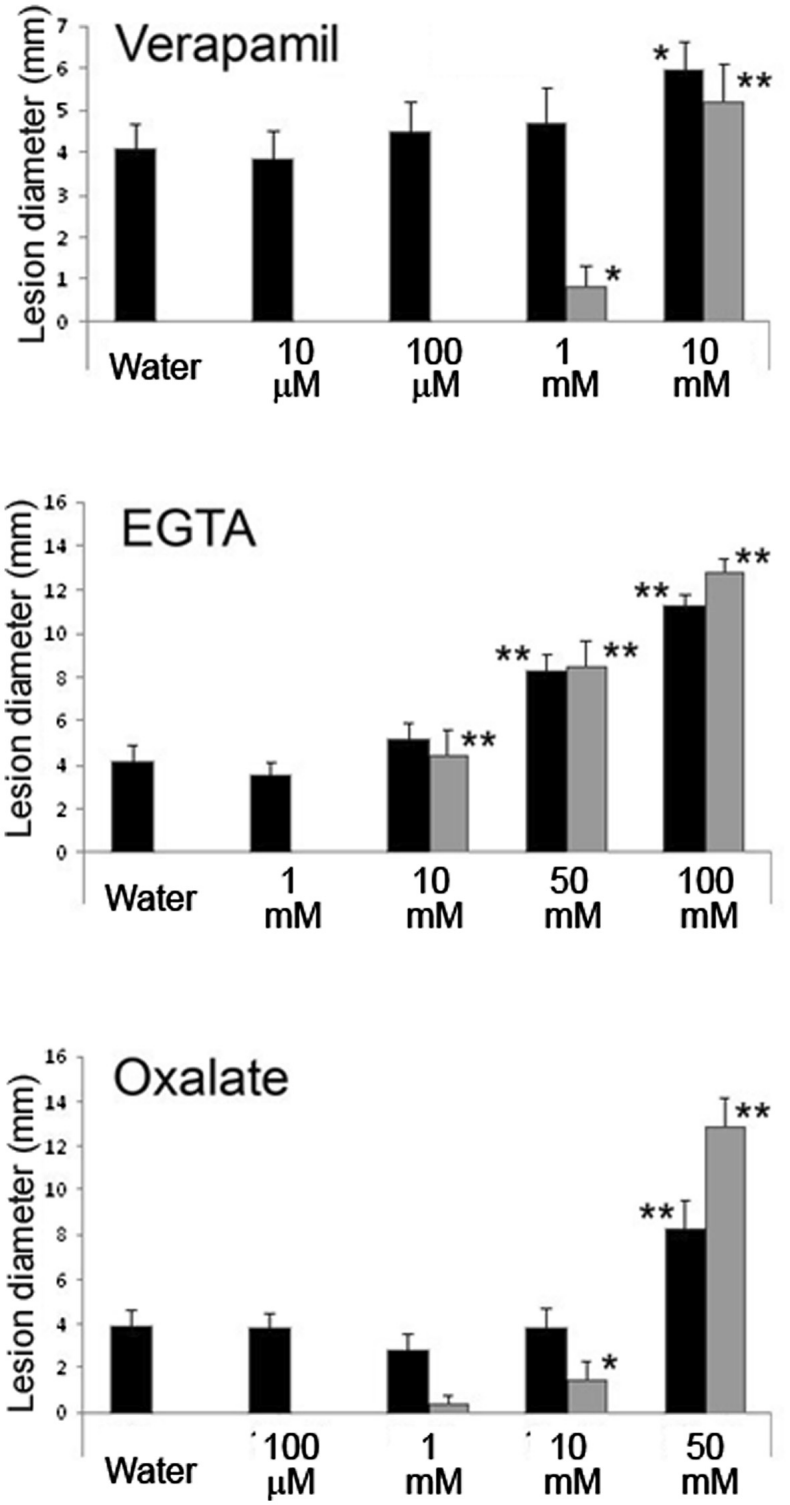
**Calcium channel blocker and calcium chelators abolish WIR.** Droplets containing different concentrations of verapamil (10 μM, 100 μM, 1 mM, 10 mM), EGTA (1 mM, 10 mM, 50 mM, 100 mM) and oxalate (100 μM, 1 mM, 10 mM, 50 mM) were applied on leaves for 3 hours. After droplet removal, leaves were wounded to induce WIR and inoculated with *B. cinerea* spores on the same site where inhibitors were applied. After three days of incubation in covered trays, lesion diameters were measured. Black: non-wounded leaves. Grey: leaves wounded right after inhibitors removal to induce WIR. Asterisks represent significant differences using Student's *t* test relative to water-treated control; *P < 0.05, **P < 0.01.

A distinctive feature of WIR is that wounded *A. thaliana* leaves become resistant to the necrotrophic fungus *B. cinerea*[5]. Thus, we tested if verapamil and chelating agents affected WIR to *B. cinerea*. Droplets of tested molecules were applied on leaves and after 3 hours, droplets were removed, leaves were wounded and suspensions of *B. cinerea* spores were placed on the wound sites. After 3 days of incubation, the wounded control was fully protected while unwounded water-treated controls displayed the typical symptoms of *B. cinerea* infection (necrosis at the infection site) (Figure 2). Similar to their effect on wound-induced ROS formation, verapamil (starting at 1 mM), EGTA (starting at 10 mM) and oxalate (starting at 10 mM) all affected WIR (Figure 2). Plants treated with oxalate and EGTA without wounding showed an enhanced lesion size, indicating a possible involvement of calcium in the basal resistance (Figure 2). Taken together, these results indicate that calcium might be involved in the pathway that couples perception of wounding with the induction of ROS and WIR.

### Cytosolic calcium concentration increases after wounding in the same cells that later display ROS burst

**Figure 3:**
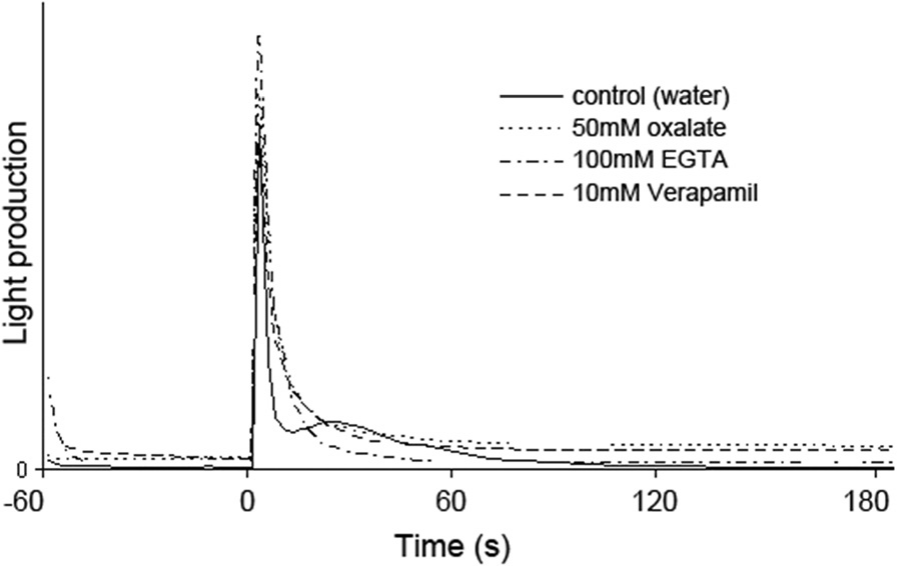
**Calcium is accumulated in the cytosol after wounding.** Leaves that constitutively express cytosolic aequorin are incubated over-night in 10 μM CTZ, placed into a luminometer and then wounded. Luminescence was measured and depended on the amount of calcium that binds to aequorin and thus reflects cytosolic calcium influx. In the control, leaves were infiltrated with water, otherwise with the mentioned concentrations of calcium inhibitors.

To further examine if calcium signalling is involved in ROS accumulation upon wounding, we tested if wounding induces changes in the cytosolic calcium concentrations using transgenic *A. thaliana* plants expressing the photoprotein aequorin [8]. Basal level and stability of the luminescence signal before wounding were then assessed by introducing the leaves in a luminometer where luminescence was immediately scored every 3 seconds for one minute. After this time, leaves were wounded directly in the luminometer and readings were carried out for three more minutes. In water-infiltrated leaves, wounding resulted in reproducible kinetics composed of a strong and transient signal between 0 and 6s that was coincident with the wounding event (Figure 3). This first peak was followed by a second transient increase of lesser amplitude, taking place after around 25–30 seconds. This second peak of luminescence was abolished in inhibitor-treated leaves compared to controls.

**Figure 4:**
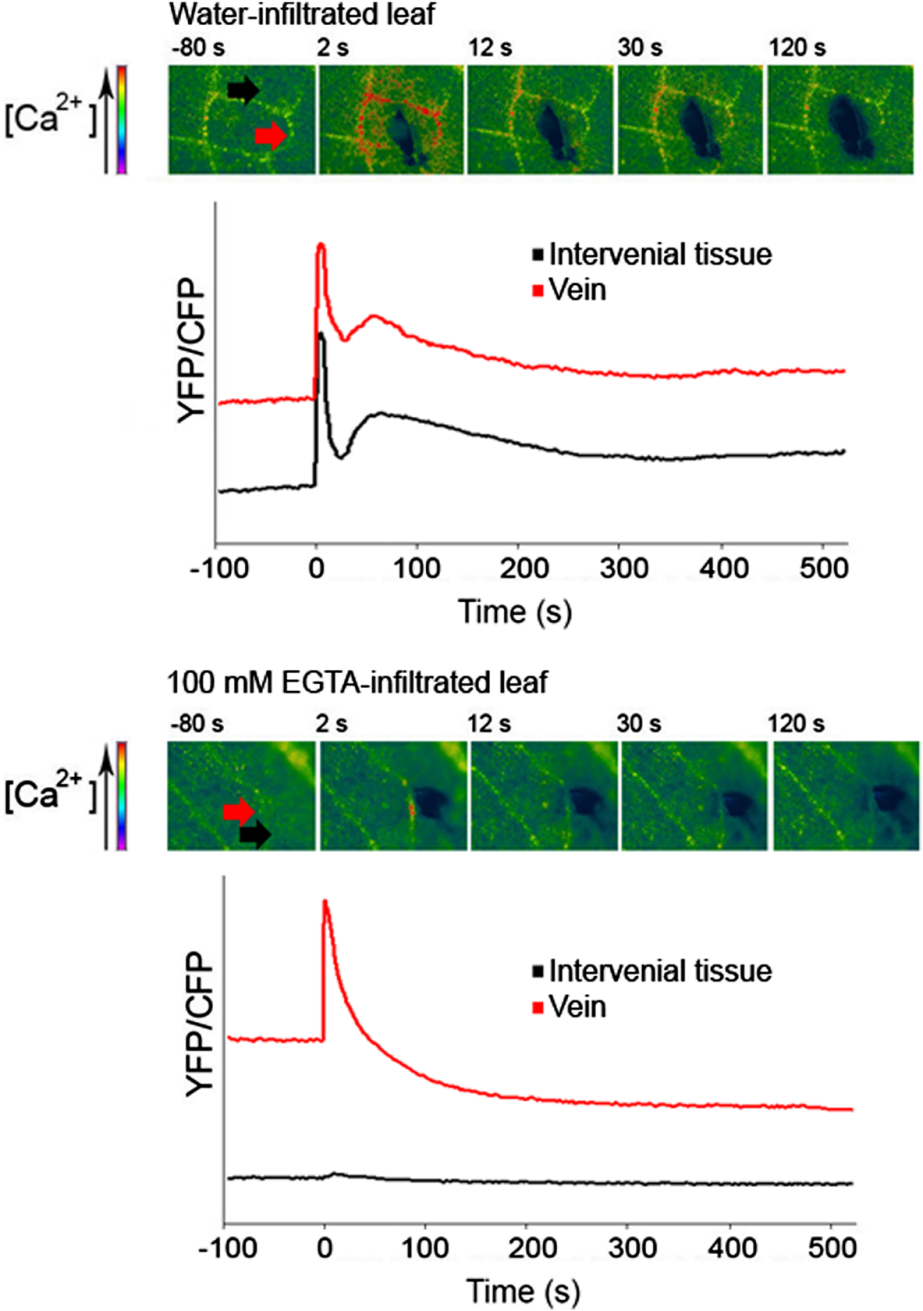
**Kinetics of cytosolic calcium influx after wounding.** Calcium appearance in cytosol of *A. thaliana* leaves after wounding was measured with plants expressing Cameleon YC3.6 infiltrated with water or with 100 mM EGTA. Upper panels: microscope images display key time points on the time course. Lower panels: kinetics resulting from the quantification of the fluorescent signals (black: interveinal tissue, red: vein). The experiments were repeated five times giving comparable kinetics, one representative time-course is presented.

**Figure 5:**
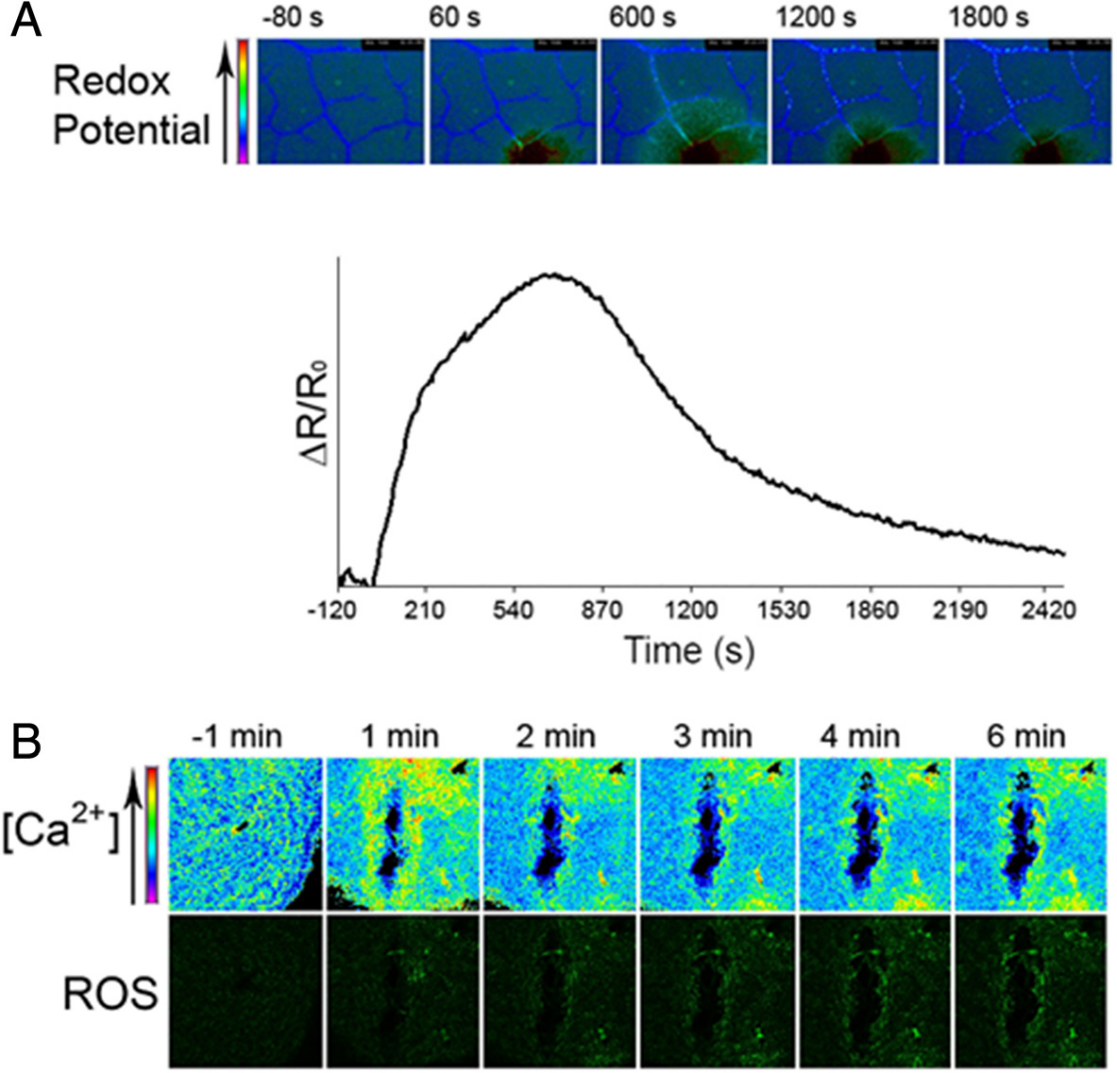
**ROS and calcium accumulate in the same cells with different kinetics. (A)** Redox potential after wounding of *A. thaliana* leaves is measured with roGFP2 expressing plants. Upper panels: microscope images display key time points on the time course. Lower panels: kinetics resulting from the quantification of the fluorescent signals. **(B)** Leaf discs obtained from plants constitutively expressing YC3.6 protein were infiltrated with DCF-DA and gently wounded with a yellow tip. FRET efficiency (upper panel) and ROS formation (lower panel) were scored in a time course with a confocal microscope taking a set of pictures every minute. The experiments were repeated 10 times giving comparable kinetics, one representative time-course is presented.

Changes in intracellular calcium were further examined using an *A. thaliana* line expressing the Yellow Cameleon 3.6 fluorescence resonance energy transfer-based Ca^2+^ sensor in the cytosol [9,10]. Wounding resulted in a first transient and steep increase in the YFP/CFP ratio (from 0 to 2s after wounding), followed by a second, smaller but more sustained one (ca 30s after wounding) (Figure 4). The increase in calcium was restricted to cells proximal to the wound site and was observed in cells composing both epidermis and vascular tissue. Both calcium peaks were abolished in the interveinal tissue of the epidermis when leaves were treated with 100 mM EGTA (black lines and arrows). Surprisingly, only the second calcium peak was abolished in the veins of EGTA-treated leaves (Figure 4). The use of plants expressing the redox sensitive biosensor roGFP2 [11] allowed also to monitor the effect of wounding on the cytosolic redox potential. Wounding resulted in an immediate increase of probe oxidation in cell neighbouring damaged tissue (Figure 5A), reflecting an effect clearly related to the observed production of ROS (Figure 1). The oxidation of roGFP2 reached a maximum after 10–12 minutes after wounding (Figure 5A). After this time it was possible to observe a slower decrease of probe oxidation, probably due to the fact that the damaged leaf is not kept in humid conditions due to equipment limitations. An additional effort was made to co-localize the calcium signal with the generation of ROS after wounding. Leaf discs of YC3.6-expressing leaves were infiltrated with the ROS-sensitive probe DCF-DA prior to observation with confocal laser scanning microscope. The time course of changes in calcium and ROS after wounding is presented in Figure 5B. Wounding lead to a rapid emission of a calcium signal at the wound site (in yellow-red, upper part Figure 5B) that decreased rapidly with time. The appearance of fluorescence (in green, lower part Figure 5B) caused by the sensing of ROS by the DCF-DA probe was also localized at the wound site but took place after the calcium signal. These results indicate that changes in calcium appear earlier than ROS and both occur at the wounding site.

## Discussion

The implication of calcium as an early signal to abiotic and biotic stresses has been previously reported [12-16]. Here we have focused on the importance of calcium in relation to WIR in *A. thaliana* leaves. Wounded leaves produce a rapid and local burst of ROS and display a strong resistance when inoculated with *B. cinerea*[5]. We have examined if changes in calcium levels are causally associated with ROS and WIR.

Our pharmacological approach using verapamil, EGTA or oxalate indicated a correlation between interference with calcium levels and loss of wound-induced ROS formation (Figure 1). These chemicals are known to act on calcium homeostasis and their application on leaves prior to wounding also inhibited WIR to *B. cinerea* (Figure 2). Overall, there was a correspondence between the concentrations at which these inhibitors interfered with ROS and those at which WIR was induced. Calcium is also required for basic resistance as non-wounded leaves treated with calcium inhibitors were also more susceptible to pathogen attack (Figure 2). The calcium channel blocker and chelating agents interfered with the early steps taking place after wounding and did not damage cells as indicated by the vital staining (Figure 1). Our results imply that calcium acts as an intermediary signal between the perception of wounding and WIR to *B. cinerea*. This conclusion supports previous observations on the importance of calcium in the process on induced resistance [13,17].

But where is the effect of calcium exactly taking place? Wounding of leaves leads to an almost immediate crushing of cells with a loss of their integrity and compartmentalization. The calcium signalling for ROS formation and induction of WIR takes place in cells adjacent to the wound site [5]. This would imply an almost instantaneous transmission of information from the crushed cells to their neighbours. Jasmonates are rapidly produced and translocated from cell to cell and across the plant [18]. Although the experimental set-up used here is similar as in the report by Glauser et al. [18], jasmonates are unlikely to be involved, since mutants affected in jasmonate synthesis still express WIR [4]). Rapid signalling from the crushed cells to their neighbours might possibly be relayed by calcium itself: decompartmentalization after wounding leads to an instantaneous and local increase in calcium that will extend from the cytosol into the apoplasm. Verapamil or calcium chelators would act in preventing the calcium wave originating at the wounded sites to enter the intact neighbouring cells.

Is it possible to visualize changes in calcium levels in leaves after wounding? We have used plants expressing aequorin or Yellow Cameleon 3.6 proteins. Both types of reporter lines indicate changes in cytosolic calcium exclusively. Using both reporters it was possible to obtain reproducible kinetics of cytosolic calcium oscillations after wounding. A sharp peak occurred in the first seconds after wounding. This peak was transient and lasted only a few seconds. The second peak appeared around 30 seconds after wounding and it lasted ca 30 seconds longer than the first one (Figure 4). This calcium signature is difficult to interpret since it represents a view of a piece of tissue rather than single isolated cells. In fact, inhibitors or calcium chelators differently affected leaf tissues. Treatment with 100mM EGTA blocked both calcium spikes caused by wounding in the interveinal tissue, but it prevented exclusively the second peak in the veins (Figure 4). These results are in line with the observations using aequorin-expressing plants, where only the second peak is prevented by treatments with inhibitors or chelators of calcium (Figure 3). Aequorin-expressing plants are a much less precise method for our purposes: to obtain a detectable signal an entire leaf wounded on a wide surface should be analyzed and both interveinal tissue and veins are indiscriminately included in the quantification. The luminescence values read during this experiment are thus a sum of the single kinetics derived by the individual tissues. More importantly, aequorin is known to respond non-linearly in certain conditions and if the concentration of calcium is not homogeneous in the sampled population of cells, the overall aequorin light emission is dominated by the most responding sub-population [19]. For those reasons the kinetics of calcium obtained by the Cameleon-expressing plants are more reliable and delimited to specific regions of interest giving a more precise idea of calcium dynamics after wounding. Both peaks are associated with the ROS formation and induction of WIR and their sensitivity to calcium inhibitors that act outside the cell would imply that calcium transits through the apoplasm.

Do the same group of cells that exhibit changes in calcium levels also produce a burst of ROS? FRET experiments combined with the introduction of the ROS marker DCF-DA showed changes in calcium levels in cells surrounding the wounded area accompanied by change in green fluorescence indicating a burst of ROS (Figure 5B). After the wound stimulus, the fluorescence caused by increased calcium levels was followed within minutes with the fluorescence reflecting increases in ROS, indication a) a colocalization of both processes and b) calcium changes precede ROS accumulation. These results obtained with cells embedded in the leaf are in agreement with many observations on cultured cells where calcium peaks preceded ROS [17]. Our results on calcium changes preceding the formation of ROS at the same site are thus in agreement with our hypothesis. It is also interesting to note, that besides transient calcium and ROS production, the oxidative status of the cells surrounding the wounding site changes, most likely caused by the burst in ROS. These conditions could cause oxidation of various molecules, for example reactive cysteines present in the cell, hence potentially triggering other downstream responses [20]. It was previously reported that a basal level of glutathione is required for WIR to *B. cinerea*[4]. After a wounding event glutathione does not further accumulate in a Col-0 wild type, but wounding leads to the priming of the expression of a glutathione-S-transferase (*GST1*) gene. These results suggest that glutathione functions in detoxification during WIR rather than building up a reduction potential that might interfere with a ROS-driven cellular oxidation. However, given the cytotoxicity of a strong ROS burst, it cannot be excluded that glutathione might be still quench molecules arising form the oxidative burst. The role of ROS in WIR is not clear at the moment. Reports indicate a role of ROS in signalling [21], while others propose a model by which ROS play a role in cell wall reinforcing [22]. We have already demonstrated that ROS are essential in WIR against *B. cinerea*[5].

What sensory proteins are possible targets for calcium? Calcium is a second messenger known to activate four different categories of calcium sensor proteins: *ca*l*m*odulins (CaMs), *C*a*M*-*l*ike proteins (CMLs), *c*alcium-*d*ependent *p*rotein *k*inases (CDPKs) and *c*alcineurin *B*-*l*ike proteins (CBLs) [23,24]. Among the 34 CDPKs of the Arabidopsis genome [25], CDPK6 was recently reported to be involved in elicitor regulated ROS and innate immunity in protoplasts of *A. thaliana*[26]. In potato CPK6, the ortholog of *A. thaliana* CPK5, can directly phosphorylate the membrane-bound NADPH oxidoreductase RBOH-D to stimulate its activity for ROS production in response to pathogens [27]. However, our previous study has already excluded an involvement of RBOH-D in WIR [5]. Here, we have tested *cpk5*, *cpk6* and *cpk11*, single, double and triple mutants [26], but WIR was still active and the mutants were perfectly able to produce ROS after wounding (data not shown). Further work is now needed to identify components involved in sensing calcium.

## Conclusions

In summary, this work demonstrates the importance of calcium as an early messenger of wound-induced signalling that leads to resistance to *B. cinerea*. Transient changes in calcium concentrations take place in a few seconds after wounding and are followed in the same location by a burst of ROS and changes in cellular oxidation. This highlights the significance of the production by *B. cinerea* of oxalate as an effector that interferes with calcium.

## Methods

### Plant maintenance

*Arabidopsis thaliana* seeds ecotype Columbia (Col-0) were grown on a pasteurized soil mix of humus and Perlite (3:1). Seeds were kept at 4°C for two days and then transferred to the growth chamber. Plants were grown in a 12 h light/12 h dark cycle with 60–70% of relative humidity, with a day temperature of 20–22°C and a night temperature of 16–18°C. WT plants were obtained from the Nottingham Arabidopsis Stock Center (Nottingham, UK).

### Culture of *B. cinerea*, inoculation, staining of hyphae, wounding procedure

*B. cinerea* strains BMM, provided by Brigitte Mauch-Mani (University of Neuchâtel, Switzerland), were grown on Difco (Becton Dickinson, http://www.bd.com) potato dextrose agar (39 g l^-1^). Spores were harvested in water and filtered through glass wool to remove hyphae. Droplets of 6 μl spore suspension (5×10^4^ spores ml^-1^) were deposited on leaves of 4-week-old plants for quantification of lesions size (mm) after 3 days. The inoculated plants were kept under high humidity in covered trays. Leaves were wounded by gently pressing the lamina with a laboratory forceps. Inoculation with *B. cinerea* was performed within 10 min after wounding, by placing a droplet of spores on the wound site. Fungal structures and dead plant cells were stained by boiling inoculated leaves for 5 min in a solution of alcoholic lactophenol trypan blue. Stained leaves were extensively cleared in chloral hydrate (2.5 g ml^-1^) at room temperature by gentle shaking, and then observed using a Leica DMR microscope with bright-field settings.

### Calcium channel inhibitors and calcium chelators treatments

Treatments with verapamil (Sigma-Aldrich), EGTA and oxalate were performed by application of a 6 μl droplet containing the inhibitors of leaves surface. All the chemical compounds were dissolved in water and the different concentrations were made by serial dilutions, water was used as control. When calcium homeostasis inhibitors were infiltrated, leaves were placed in a 50 ml syringe tubes without needle filled with 10 ml of solution containing the appropriate inhibitor, the cap was then blocked and the piston was gently pulled 5 times.

### Detection of ROS

ROS were detected using the fluorescent probe 5-(and 6)-carboxy-2′,7′-dichloro dihydrofluorescein diacetate (DCF-DA) (Sigma-Aldrich). Wounded or unwounded leaves were vacuum-infiltrated (3×3 min) in 60 μM of DCF-DA in a standard buffer (1 mM KCl, 1 mM MgCl_2_, 1 mM CaCl_2_, 5 mM 2-morpholinoethanesulfonic acid adjusted to pH 6.1 with NaOH). Leaves were then rapidly rinsed in DCF-DA buffer and observed using a Leica DMR epifluorescence microscope with a GFP filter set (excitation 480/40 nm, emission 527/30 nm) (Leica). Microscope images were saved as TIFF files and processed for densitometric quantification with Image J version 1.44 (NIH). Software settings were kept the same for every image analyzed within one experiment, background values of 2 to 6 were subtracted from each image before processing. For co-localization of ROS and calcium, ROS were stained with DCF-DA and visualized using an excitation light at 488 nm (470/40 nm) and an emission 525/50 nm for FITC/GFP with a dichroic mirror 500 nm.

### Detection of calcium

The monitoring of changes in cytosolic calcium concentrations was performed using transgenic *A. thaliana* plants expressing aequorin under the control of the cauliflower mosaic virus promoter 35S (gift from Marc Knight, Durham University). Leaves from 4 weeks-old aequorin-expressing plants were incubated overnight in 10 μM of coelenterazine (CTZ) in the dark to allow the binding between CTZ and aequorin. After overnight incubation in CTZ, leaves were gently manipulated by the petiole and vacuum-infiltrated with calcium inhibitors or water as control and left untouched for 5 min. Basal level and stability of the luminescence signal before wounding were then assessed by introducing the leaves in the luminometer (Sirius, Berthold Detection System) where luminescence values were immediately scored every 3 s for one min. After this time, leaves were wounded directly in the luminometer and reading was carried out for 3 min. Experiments have been repeated 10 times and one representative result is shown.

### Time-lapse calcium and roGFP2 imaging

Whole leaves of 16 to 21 day-old plants expressing cytosolic localized Cameleon YC3.6 [9] or expressing the roGFP2 redox sensor [11] were placed in an open top chamber. Leaves were imaged *in vivo* by an inverted fluorescence microscope Nikon Ti-E (Nikon, JP, http://www.nikon.com/) with CFI planfluor 4x A.N.0,13 dry objective. Excitation light was produced by a fluorescent lamp Prior Lumen 200 PRO (Prior Scientific, UK) at 440 nm (436/20 nm) for Cameleon or switching between 470/40 nm and 405/40 nm for roGFP2. Images were collected with a Hamamatsu Dual CCD Camera ORCA-D2 (Hamamatsu, Photonics, JP). For Cameleon analysis the FRET CFP/YFP optical block A11400-03 (Emission 1 483/32 nm for CFP and Emission 2 542/27 nm for cpVenus with a dichroic mirror 510 nm) (Hamamatsu, Photonics, JP) was used for the simultaneous CFP and cpVenus acquisitions. Exposure time was 400 ms with a 2x2 CCD binning and images where acquired every 2 sec. The roGFP2 emissions were collected with a bandpass filter of 505–530 nm for both excitation wavelengths. Exposure time was 30 ms with a 2x2 CCD binning for the 470/40 nm excitation, and 300 ms with a 2x2 CCD binning for the 405/40 nm excitation and images where acquired every 5 sec. Filters and dichroic mirror were purchased from Chroma (Chroma Technology Corporation, USA). The NIS-Elements (Nikon, JP) was used as platform to control microscope, illuminator, camera and post-acquisition analyses. With respect to time course experiments, fluorescence intensity was determined over regions of interest (ROIs) that correspond to the region surrounding the wounded site. The wounding was made with forceps. Since imaged areas do not contain any background, the latter was not subtracted. For Cameleon analysis cpVenus and CFP emissions of the analyzed ROIs were used for the ratio (R) calculation (cpVenus/CFP) and plotted versus time. For roGFP2 analysis the two emissions of the analyzed ROIs were used for the ratio (R) calculation (405/488) and normalized to the initial ratio (R0) and plotted versus time (ΔR/R0). Experiments have been repeated 10 times and one representative result is shown.

## Competing interests

The authors declare that they have no competing interests.

## Authors’ contributions

EB: ROS staining and quantification, trypan blue staining, *B. cinerea* infections. AC: time-lapse calcium and roGFP2 imaging, manuscript preparation. FL: conceptualization of the experiments, critical revision of the manuscript. JPM: conceptualization of the experiments, manuscript writing, critical revision of the manuscript. MB: conceptualization of the experiments, calcium influx kinetics using aequorin expressing plants, ROS and calcium co-staining, manuscript writing. All authors read and approved the final manuscript.

## Supplementary Material

Additional file 1 **Infiltration of wounded *A. thaliana* leaves with calcium channel blockers and calcium chelators abolishes ROS production.** Different concentrations of verapamil, EGTA and oxalate were vacuum-infiltrated into detached leaves. Leaves were then wounded and stained with DCF-DA and visualized with a fluorescence microscope (ROS in green and chlorophyll autofluorescence in red). Densitometric analysis of the ROS signal is displayed on the right of each image series. Asterisks represent significant differences using Student's *t* test relative to water-treated control; *P < 0.05, **P < 0.01. Click here for file
