# Supplementary figures and images for: Production of reactive oxygen species and wound-induced resistance in Arabidopsis thaliana against Botrytis cinerea are preceded and depend on a burst of calcium
Source: BMC Plant Biol. 2013 Oct 17;13:160. doi: 10.1186/1471-2229-13-160 (PMC4016300; doi:10.1186/1471-2229-13-160)

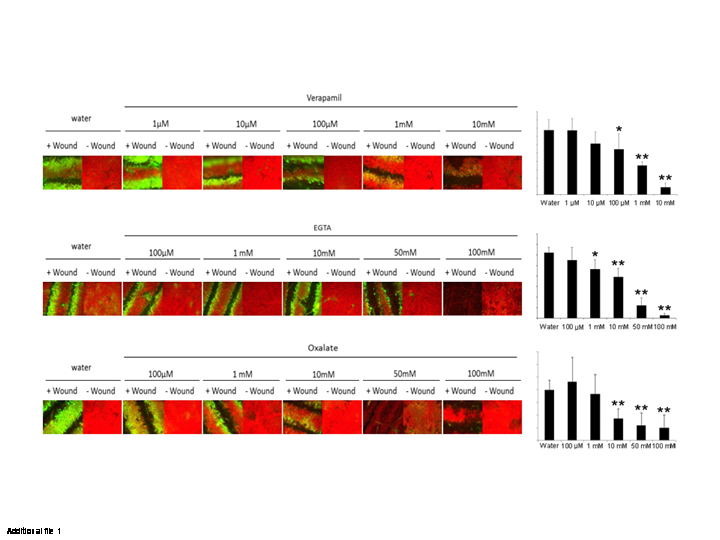

Supplement: Additional file 1 — Infiltration of wounded A. thaliana leaves with calcium channel blockers and calcium chelators abolishes ROS production. Different concentrations of verapamil, EGTA and oxalate were vacuum-infiltrated into detached leaves. Leaves were then wounded and stained with DCF-DA and visualized with a fluorescence microscope (ROS in green and chlorophyll autofluorescence in red). Densitometric analysis of the ROS signal is displayed on the right of each image series. Asterisks represent significant differences using Student's t test relative to water-treated control; *P < 0.05, **P < 0.01. [file 1471-2229-13-160-S1.tiff]
